# Examining Form and Function of Dendritic Spines

**DOI:** 10.1155/2012/704103

**Published:** 2012-04-17

**Authors:** Kevin F. H. Lee, Cary Soares, Jean-Claude Béïque

**Affiliations:** Heart and Stroke Foundation Centre for Stroke Recovery, Centre for Neural Dynamics, and, Department of Cellular and Molecular Medicine, University of Ottawa, 451 Smyth Road, Rm 3501N, Ottawa, ON, Canada K1H 8M5

## Abstract

The majority of fast excitatory synaptic transmission in the central nervous system takes place at protrusions along dendrites called spines. Dendritic spines are highly heterogeneous, both morphologically and functionally. Not surprisingly, there has been much speculation and debate on the relationship between spine structure and function. The advent of multi-photon laser-scanning microscopy has greatly improved our ability to investigate the dynamic interplay between spine form and function. Regulated structural changes occur at spines undergoing plasticity, offering a mechanism to account for the well-described correlation between spine size and synapse strength. In turn, spine structure can influence the degree of biochemical and perhaps electrical compartmentalization at individual synapses. Here, we review the relationship between dendritic spine morphology, features of spine compartmentalization and synaptic plasticity. We highlight emerging molecular mechanisms that link structural and functional changes in spines during plasticity, and also consider circumstances that underscore some divergence from a tight structure-function coupling. Because of the intricate influence of spine structure on biochemical and electrical signalling, activity-dependent changes in spine morphology alone may thus contribute to the metaplastic potential of synapses. This possibility asserts a role for structural dynamics in neuronal information storage and aligns well with current computational models.

## 1. Introduction

Ever since the first description of *espinas* on Purkinje cells by Cajal more than 100 years ago [1, 2], these tiny, femtolitre-sized, structures have been found on dendrites of a wide variety of neuronal cell types and the role of these minute structures in neuronal function has been the subject of considerable attention, speculation, and debate. These discrete dendritic protrusions form a rich structural scaffold for the majority of excitatory synapses in the brain, harbouring a complement of biochemical signalling machinery as well as a proteinaceous postsynaptic density (PSD) containing, amongst others, ionotropic glutamate receptors of the AMPA and NMDA subtypes [3]. These receptors mediate the bulk of fast excitatory neurotransmission in the brain. During postnatal development, dendritic spines acquire AMPARs and undergo structural enlargement, resulting in a positive correlation between spine size and AMPAR function. Interestingly, the high degree of heterogeneity in dendritic spine structure and function at maturity suggests that spine growth is regulated in a synapse-specific manner and not simply a consequence of *en masse *spine development.

In the past decade or so, a number of technological developments in fluorescence microscopy and molecular techniques have greatly accelerated our understanding of the relationship between structure and function at dendritic spines. For one, the induction of synaptic plasticity at single synapses was found to result in changes in spine structure, providing a plausible mechanism to explain the concurrent developmental changes in spine size and function [4]. Furthermore, recent studies have elaborated a mechanistic and molecular framework to suggest that spines function as discrete compartments, offering a basis for computationally relevant synaptic autonomy. Based on the robust concordance between structural and functional plasticity, and on the similarities in the molecular underpinnings that drive these two processes, there is a growing trend in synaptic physiology to infer synaptic strength based on characteristics of spine morphology. However, the dissociation of spine structure and function under some experimental conditions suggests an important mechanistic divergence in the regulation of spine form and function. In this paper, we will provide an outline of the dendritic spine as a discrete functional compartment, discuss new developments in structural and functional plasticity at single spines and highlight key aspects of our understanding of the relationship between spine structure and function.

## 2. Two-Photon Microscopy and the Investigation of Individual Dendritic Spines

Despite unsheathing fundamental properties of various forms of synaptic plasticity [5], investigations based solely upon electrically evoked synaptic events left a number of open questions. Although minimum stimulation methods allow the functional study of single synapses in isolation [6–9], the inherent technical challenges of these experiments hinder the ability to efficiently amass data and resolve spatial parameters such as the morphology and location of the activated synapses (relative to each other and to the soma). The advent of two-photon (2P) laser scanning microscopy circumvented a number of these experimental limitations and has contributed considerable depth to our understanding of spine function and plasticity.

The longer wavelengths and lower excitation energy used in 2P imaging increase imaging depth in scattering tissue (such as brain) while also reducing photodamage/toxicity compared to 1P imaging [10–12]. Furthermore, the 2P excitation event is highly restricted in physical space with an excitation volume that roughly approximates the diffraction limits of the optical system [10]. This small excitation volume thus confers the ability to photoactivate molecules with high spatial precision, thereby providing novel opportunities for the study of synaptic physiology. For instance, 2P “uncaging” of caged forms of neurotransmitters (for, e.g., MNI-Glutamate) provides the ability to selectively activate spatially discrete glutamate receptors in a number of experimental preparations *in vitro* [4, 13–24] and *in vivo* [25]. Pioneering work by Matsuzaki and colleagues used 2P imaging and glutamate uncaging to probe AMPAR content and induce LTP at individual dendritic spines on hippocampal CA1 pyramidal neurons, generating key insight into single synapse plasticity [4, 22]. For instance, the induction of LTP at single dendritic spines via 2P glutamate uncaging circumvented the presynaptic component of synaptic transmission and provided unequivocal support to the notion that, at least under certain conditions, synaptic plasticity can be mediated by solely postsynaptic mechanisms [4, 26].

In addition to providing important information regarding plasticity at single synapses, advances in 2P imaging and related optical techniques have been instrumental in generating novel understanding of other neuronal mechanisms and properties such as the spatial distribution of synaptic weights, the autonomy of the spine as a functional compartment, the integrative behaviour of dendritic branches, and the recurrent connectivity of cortical circuits [14, 16, 21, 27].

## 3. A Compartmental Model of Dendritic Spines

Dendritic spines are specialized structures exhibiting a high degree of molecular organization and exist in a wide range of morphologies. Although a number of nomenclatures have been proposed to describe the breadth of morphologies that individual spines can adopt, they can be broadly summarized as follows: “Mushroom-like,” identified by a round dendritic spine head connected to the parent dendrite by a spine neck; “Stubby” spines, which are short, stout protrusions or bumps with no definitive spine neck; filipodial/long spines, which appear as thin, finger-like protrusions [28, 29]. There has been considerable speculation on the specific role imparted by these varying morphologies on aspects of spine function. For one, substantial attention has been given to the role of the spine neck and accumulating experimental evidence suggests that it serves to compartmentalize the dendritic spine head. This compartmental model is particularly attractive in light of the synapse specificity of the structural and functional changes that take place over development and during plasticity. The compartmentalization of dendritic spines can be broadly divided into two functional domains: (i) the biochemical compartment, which describes the spatial confinement of biochemical signalling due to diffusional restriction and physical segregation of proteins and signalling molecules; (ii) the electrical compartment, where spine neck morphology can impact the kinetics and propagation of synaptic potentials in a spine-specific manner. Here, we will sequentially review these two functional domains.

### 3.1. The Biochemical Compartment

**Figure 1:**
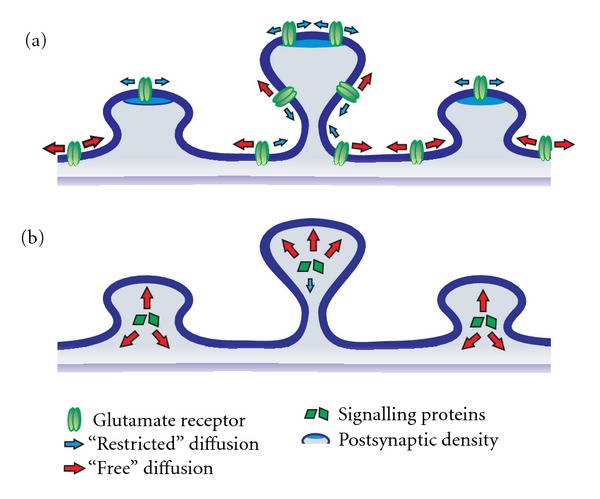
*Biochemical compartmentalization in dendritic spines.* The spine neck may offer enhanced compartmentalization of biochemical signalling at synapses. (a) The lateral mobility of surface glutamate receptors is attenuated across longer spine necks and at the postsynaptic density. (b) The spine neck imposes diffusional constraints on cytosolic signalling proteins. These mobility restraints imposed by the spine neck create spine-specific compartmentalization of cytosolic signalling and surface receptor dynamics.

Postsynaptic induction and expression of several forms of synaptic plasticity requires calcium influx through NMDARs and the initiation of calcium-dependent biochemical signalling in the dendritic spine. The development of calcium-sensitive fluorescent indicators and imaging techniques has greatly facilitated the study of calcium dynamics during synaptic activity. Specifically, calcium imaging experiments demonstrate that NMDAR-mediated calcium influx elicited during synaptic transmission is tightly restricted to the spine head, with minimal calcium diffusion into the parent dendrite [18, 30–33]. Given the key role of calcium as a second messenger in the regulation of synaptic plasticity, highly compartmentalized calcium signalling at dendritic spines is likely critical for providing the synapse specificity of synaptic plasticity. As a result, it has been proposed that the primary function of the dendritic spine structure is to compartmentalize signalling molecules such as calcium [31, 34]. Many factors can influence the intracellular diffusion of calcium. For instance, the presence of a spine neck has been suggested to restrict calcium diffusion and also appears to limit the diffusion of other molecules such as GFP and fluorescein dextran [31, 33, 35–37]. In addition, calcium pumps such as PMCAs and SERCA, calcium-binding molecules such as calmodulin (CaM) or calbindin, and differential cytosolic viscocities at individual spines can all contribute to regulate free-calcium concentrations (and its dynamics) and influence intracellular diffusion [38–40]. Together, these diverse mechanisms indicate that dendritic spines utilize multiple strategies to compartmentalize biochemical signals and promote autonomous synaptic function (see Figure 1).

Dendritic spines must also communicate with protein synthesis machinery located in the parent dendrite to sustain late phases of LTP [23, 41–43]. Thus, the movement of signalling molecules to and from the dendritic spine must not be fully compartmentalized but conforms to some degree of regulation. An illustration of such regulation is provided by recent experiments showing that calcium/calmodulin-activated kinase II (CaMKII) and Ras, two important molecules for synaptic plasticity, exhibit differential displacements from activated spines into the parent dendrites during synaptic plasticity [19, 44]. Recent work by Murakoshi et al. (2011) extended these findings using a FRET-based approach [45]. The authors assessed the spatial spread of two synaptically activated Rho-GTPases, RhoA and Cdc42. Whereas single-spine LTP induced by 2P glutamate uncaging leads to sustained activation (up to 30 min) of both RhoA and Cdc42, only activated RhoA readily traversed the spine neck into the parent dendrite, with activated Cdc42 restricted to the stimulated spine [45]. Since the measured diffusional properties of these proteins were similar, it was proposed that mechanisms localized to the spine head were likely required to spatially restrict Cdc42 activation, thereby enforcing the notion that spines are highly regulated biochemical compartments. Taken together, the spatial compartmentalization of key regulatory molecules (e.g., protein kinases) may also offer powerful constraints that impact the spread of intracellular signals from the spine to the parent dendrite.

Surface (plasma membrane-bound) AMPARs and NMDARs exist at both synaptic and extrasynaptic locations. These surface receptor populations are not rigidly fixed, but in perpetual diffusional flux laterally through the membrane [46–48]. Similar to intracellular diffusion, the lateral mobility of proteins in the plasma membrane can also be influenced by morphological parameters of spines [49–51]. For instance, FRAP analysis demonstrated that spine necks restrict the lateral diffusion of surface AMPARs. Specifically, AMPARs at spines connected to the parent dendrite by a spine neck exhibit a twofold slower rate of lateral mobility compared to those at spines without a distinguishable spine neck (Figure 1). Similar results were obtained using membrane-bound GFP, indicating that the impedance of lateral mobility was dictated by morphological parameters of the spine, and not by intrinsic properties of AMPAR trafficking *per se *[49]. Furthermore, AMPARs also undergo constitutive vesicular cycling via endo- and exocytosis. Evidence from both electron microscopy and fluorescence imaging indicates the presence of endocytic and exocytic zones within dendritic spines [52–55]. Interestingly, the dynamic balance of endo- and exocytosis modulates synaptic strength and underlie certain forms of plasticity. Indeed, LTP induction results in an enhancement of AMPAR exocytosis [56–60]. Taken together, the strategic clustering of signalling proteins, the development of narrow spine necks, and the organization of vesicular cycling machinery can all contribute to biochemical compartmentalization of spines. This compartmentalization provides individual spines with the autonomous capacity to dynamically regulate the surface expression of distinct pools of AMPARs to promote the synapse specificity of synaptic plasticity.

### 3.2. The Electrical Compartment

In addition to providing the morphological substrate for bestowing features of biochemical compartmentalization, spines may also function as electrical compartments capable of modulating the amplitude, kinetics, and integration of synaptic potentials. Early estimations based on Rallian cable theory and measurements of molecular diffusion indicated that only modest ohmic resistances would be provided by spine necks and therefore largely dismissed the notion that spines behave as electrical compartments [37, 61]. However, recent experimental evidence suggests that electrical compartmentalization can take place in at least a subset of dendritic spines [13, 17, 62]. A combination of current-clamp recordings, 2P uncaging, and second harmonic membrane potential measurements provided evidence that long spine necks attenuate synaptic potentials between spine head and the parent dendrite [13]. In this line, it is interesting to note that calcium transients induced by activation of NMDARs can be readily detected by 2P calcium imaging in physiological extracellular magnesium concentrations (~1.0 mM) in slices [63–65] and *in vivo* [66–68], despite the presence of the voltage-dependent magnesium block of NMDARs near resting membrane potentials. Furthermore, calcium transients mediated by voltage-sensitive calcium channels (VSCCs) can also be visualized upon synaptic activation, indicating unexpectedly large depolarizations at the spine head [17]. Together, these data suggest that spine necks may impart an appreciable degree of electrical resistance—and charge accumulation in spine heads—and thus electrically compartmentalize dendritic spines [62]. An intriguing and functionally powerful idea is that the degree of both electrical and biochemical compartmentalization might be dictated by active modifications in spine morphology. This possibility is becoming increasingly prominent given the dynamic structural changes which accompany the expression of synaptic plasticity (see below).

## 4. Structural and Functional Plasticity at Spines

The development of 2P glutamate uncaging to stimulate and induce LTP at single dendritic spines has been instrumental in providing key insights on the structural and functional changes that take place during plasticity. In 2004, Matsuzaki and colleagues induced LTP at individual dendritic spines by 2P glutamate uncaging and showed that the expression of LTP is associated with spine enlargement [4]. Furthermore, smaller spines carried an inherently higher propensity for LTP expression compared to larger spines, which were functionally and structurally more stable. Interestingly, some of the molecular mechanisms underlying structural plasticity have been found to closely parallel those for synaptic plasticity. For instance, LTP induction stimuli involving strong synaptic input and large postsynaptic rises in calcium facilitate actin branching and polymerization, providing a protrusive force to mediate spine enlargement [4, 69–72]. Conversely, LTD-inducing stimuli lead to actin depolymerization, spine shrinkage, and retraction [70]. Moreover, similar to the expression of long-lasting phases of LTP, the temporal stability of structural plasticity requires the synthesis of new proteins [23, 42]. These fundamental similarities in the induction of both structural and functional plasticity indicate an intimate relationship between these two processes.

One critical molecular link is CaMKII, a highly abundant protein in spines with an established role in synaptic plasticity [4, 44, 73–75]. At rest, CaMKII directly bundles and stabilizes actin filaments and is involved in the structural stability of spines [76]. CaMKII is activated by LTP-inducing stimuli, remaining persistently active and spatially compartmentalized to the stimulated spine properties that correlate well with the spatiotemporal characteristics of structural and functional plasticity [44]. Moreover, active CaMKII dissociates from the actin cytoskeleton causing structural destabilization, thus permitting structural modifications of the spine to take place [76]. Downstream, CaMKII activates a number of signalling molecules such as members of the Rho-GTPase family (RhoA, Cdc42, Rac1, and Rnd1) to mediate changes in spine structure [45, 72, 77]. For instance, Cdc42 becomes activated during LTP induction and interacts with p21-activated kinase (PAK) proteins to stabilize structural modifications [45]. Mice expressing a dominant-negative PAK (dnPAK) transgene in the forebrain show abnormal dendritic spine morphology, defects in both LTP and LTD, and impairments in the consolidation of spatial and fear memory [78]. Whereas it is difficult to attribute the behavioural deficits exhibited by dnPAK mice to synaptic impairments alone, these experimental strategies help to elucidate the interplay between structural and functional plasticity.

Although structural and functional changes rely on common signalling molecules, is it possible for these changes to occur independently of one another? Some evidence suggests that structural and functional plasticity are mutually stabilizing processes. For instance, in CA1 pyramidal neurons, the temporal stability of LTP expression is dependent on actin polymerization [79]. Subsequent investigations have expanded on these findings, underscoring a critical role for cytoskeletal actin dynamics in the directed trafficking of AMPARs [69, 70, 78, 80, 81]. Conversely, the insertion of AMPARs during LTP not only acts to increase synaptic strength, but has also been suggested to stabilize structural changes of the spine [82]. These data suggest that the molecular components that drive structural changes in dendritic spines during plasticity also act to stabilize AMPAR insertion and vice versa. This dynamic interplay thus provides a basis for the tight association between changes in spine volume and AMPAR content during LTP.

## 5. Structure versus Function

Dendritic spines on pyramidal cell dendrites number in the thousands and exhibit a high degree of morphological heterogeneity. High-resolution electron microscopy studies provided the first indication that spine form and function were related by demonstrating that the size of the PSD and number of AMPARs positively correlate with the size of spines [83–86]. A number of recent studies provided functional support to these ultrastructural findings by showing a strong positive correlation between dendritic spine size and AMPAR function (i.e., size of AMPAR-mediated current), as determined by 2P glutamate uncaging [14, 15, 22, 25, 35]. Considering the parallel changes observed in both structure (i.e., spine volume) and function (i.e., AMPAR content) during activity-dependent plasticity, it is perhaps not at all surprising that such a correlation exists. However, a more detailed and in-depth look at the literature, as outlined below, reveals that spines, at least in certain conditions, have the ability to significantly depart from such a tight structure/function coupling.

One of the first indications pointing to a divergence of spine form and function was provided by Smith et al. (2003) while describing the distance-dependent scaling of synaptic AMPARs in hippocampal CA1 pyramidal neurons [27]. Using a combination of whole-cell and dendritic recordings with 2P glutamate uncaging, they showed the synaptic weights of spines were progressively larger with increasing distances from the soma. However, this apparent increase in spine function was not accompanied with measurable changes in spine volume. Nonstationary fluctuation analysis on 2P glutamate uncaging currents further revealed that this increase in function with dendritic distance reflected a higher density of spine AMPARs, and not an enhanced single-channel conductance. Together, these data provide a convincing illustration that spines of similar size can express a strikingly wide range of AMPAR density.

A disconnect between dendritic spine structure and function is further evidenced in studies of “silent” synapses. Silent synapses contain detectable NMDARs but are devoid of AMPARs and are therefore largely “silent” at rest (ought to the magnesium-dependent blockade NMDARs at resting membrane potential). They are thought to represent immature glutamateric synapses in part because their expression drastically diminishes during postnatal development [26, 87, 88]. Not surprisingly, early 2P-glutamate uncaging investigations described the presence of silent synapses on thin, filipodial-like spines in developing CA1 pyramidal neurons [15]. Interestingly, however, subsequent investigations in the rodent somatosensory cortex reported that silent synapses can be detected at spines spanning a broad range of morphologies [14, 18]. Although providing indirect support to this notion, work in PSD-95 KO mice also documented the presence of a structure/function uncoupling for spines. At an age where silent synapses were no longer detected in WT mice, PSD-95 KO mice displayed a high proportion of silent synapses onto large spines that otherwise appeared mature [15]. Collectively, these data indicate that although there is a clear correlation between spine size and function, there is also room for a significant departure from this tight structure/function coupling.

Studies on the dynamical nature of spine structure during LTD also indicate a divergence in the signalling pathways that regulate spine size and function. For instance, Zhou et al. (2004) reported that LTD and spine shrinkage at hippocampal synapses show differential requirements for protein phosphatase signalling via PP1 and calcineurin, despite sharing a similar requirement for NMDAR activation [89]. Furthermore, while the actin-binding protein cofilin was involved in spine shrinkage, it seemed to play no role in the expression of LTD. More recent investigations have also indicated that clathrin-mediated endocytosis is not required for spine shrinkage, despite being essential for LTD expression [90]. Finally, LTD studies in cerebellar Purkinje cells also reported dissociation between spine structure and function. Indeed, Sdrulla and Linden reported that LTD at cerebellar parallel fiber-Purkinje cell synapses was not associated with changes in either spine number or volume. In an interesting twist, the authors further identified a manipulation that led to a dramatic and global retraction of spines on Purkinje neurons that, surprisingly, was not associated with significant changes in synaptic strength [91]. Thus, evidence obtained from LTD experiments in two distinct brain regions underscores a mechanistic divergence of spine structure and function.

**Figure 2:**
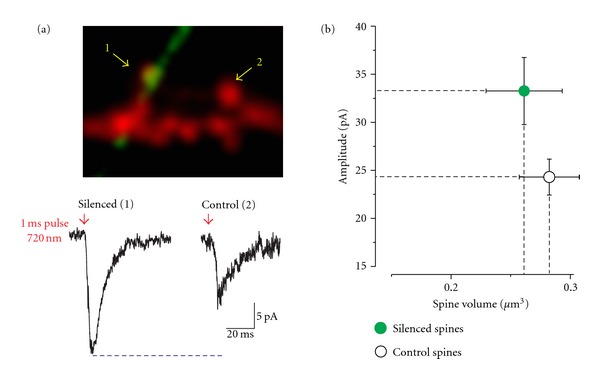
*Dissociation of spine size and synaptic strength*. (a) The release of glutamate was reduced for 48 hours specifically on the spine marked (1). This leads to a homeostatic enhancement of synaptic strength, as assessed by 2P-uncaging of MNI-Glutamate. The size of the synaptic currents induced by 2P-uncaging is shown in the bottom panel. (b) The significant enhancement of the amplitude of synaptic currents onto “silenced” spines was not accompanied by any measurable changes in spine volume. Adapted from [15].

Lastly, this divergence is further exemplified in a model of single-synapse homeostatic plasticity in dissociated cortical neuronal cultures. Homeostatic plasticity refers to the ability of a neuron to bidirectionally tune synaptic AMPAR content in response to changes in overall network activity [92]. Recent experiments have expanded these findings by showing that homeostatic regulation of synaptic strength can be achieved at the level of individual dendritic spines [16, 93]. In one experimental paradigm, chronic suppression of presynaptic glutamatergic input onto single spines leads to an enhancement of postsynaptic AMPAR function, as determined by 2P glutamate uncaging [16]. Interestingly, despite a marked increase in AMPAR currents, there were no discernable changes in spine volume (see Figure 2). By comparing the current-voltage (I-V) relationship of AMPARs at these two populations of dendritic spines, activity-deprived synapses were found to express AMPARs with a distinct subunit composition (AMPARs lacking the GluA2 subunit). Because this AMPAR subtype has an inherently higher conductance, this switch in subunit composition provides a mechanistically plausible model to account for the increased synaptic strength onto spines of similar volume.

## 6. Conclusion

As a major component of excitatory synapses, spines are strategically poised to support important modulatory roles in synaptic transmission and neuronal function. Although still subject to debate, an emerging notion posits that spines provide a structural scaffold to act as biochemical and electrical compartments. Interestingly, discrete differences in dendritic spine morphology may directly influence the degree of functional compartmentalization (Figure 1).

In addition, the dynamic nature of spine structure [94, 95] may generate parallel changes in the compartmentalization features of individual spines. One can speculate that these morphologically dependent degrees of compartmentalization lead to distinct states of metaplasticity at individual synapses. This notion aligns well with emerging theoretical models of synaptic learning that demonstrate that synapses exhibiting a gradation of states, each bridged by distinct metaplastic transitions, bestow neural networks with enhanced information storage capacity [96, 97]. Altogether, these considerations highlight the rich computational potential afforded by the yet to be completely understood relationship between form and function of dendritic spines.
